# Inter-individual differences in foveal shape in a scavenging raptor, the black kite *Milvus migrans*

**DOI:** 10.1038/s41598-020-63039-y

**Published:** 2020-04-09

**Authors:** Simon Potier, Mindaugas Mitkus, Thomas J. Lisney, Pierre-François Isard, Thomas Dulaurent, Marielle Mentek, Raphaël Cornette, David Schikorski, Almut Kelber

**Affiliations:** 1 Department of Biology, Lund University, Sölvegatan 35, Lund, S-22362 Sweden; 2 Institute of Biosciences, Life Sciences Center, Vilnius University, Vilnius, Lithuania; 3 CEFE UMR 5175, CNRS - Université de Montpellier - Université Paul-Valéry Montpellier - EPHE, Montpellier, France; 4 Unité d’Ophtalmologie, Centre Hospitalier Vétérinaire, Saint-Martin-Bellevue, France; 5 Institut de Systématique, Evolution, Biodiversité (ISYEB) - Muséum National d’Histoire Naturelle, CNRS, Sorbonne Université, EPHE, Université des Antilles, 57 rue Cuvier, CP 50, 75005 Paris, France; 6 Laboratoire Labofarm, Loudeac, France

**Keywords:** Animal physiology, Retina

## Abstract

Birds, and especially raptors, are believed to forage mainly using visual cues. Indeed, raptors (scavengers and predators) have the highest visual acuity known to date. However, scavengers and predators differ in their visual systems such as in their foveal configuration. While the function of the foveal shape remains unknown, individual variation has never been quantified in birds. In this study, we examined whether foveal shape differs among individuals in relation to eye size, sex, age, eye (left or right) and genetic proximity in a scavenging raptor, the black kite *Milvus migrans*. We assessed foveal shape in 47 individuals using spectral domain optical coherence tomography (OCT) and geometric morphometric analysis. We found that foveal depth was significantly related to eye size. While foveal width also increased with eye size, it was strongly related to age; younger individuals had a wider fovea with a more pronounced rim. We found no relationship between foveal shape and genetic proximity, suggesting that foveal shape is not a hereditary trait. Our study revealed that the shape of the fovea is directly linked to eye size and that the physical structure of the fovea may develop during the entire life of black kites.

## Introduction

**Figure 1:**
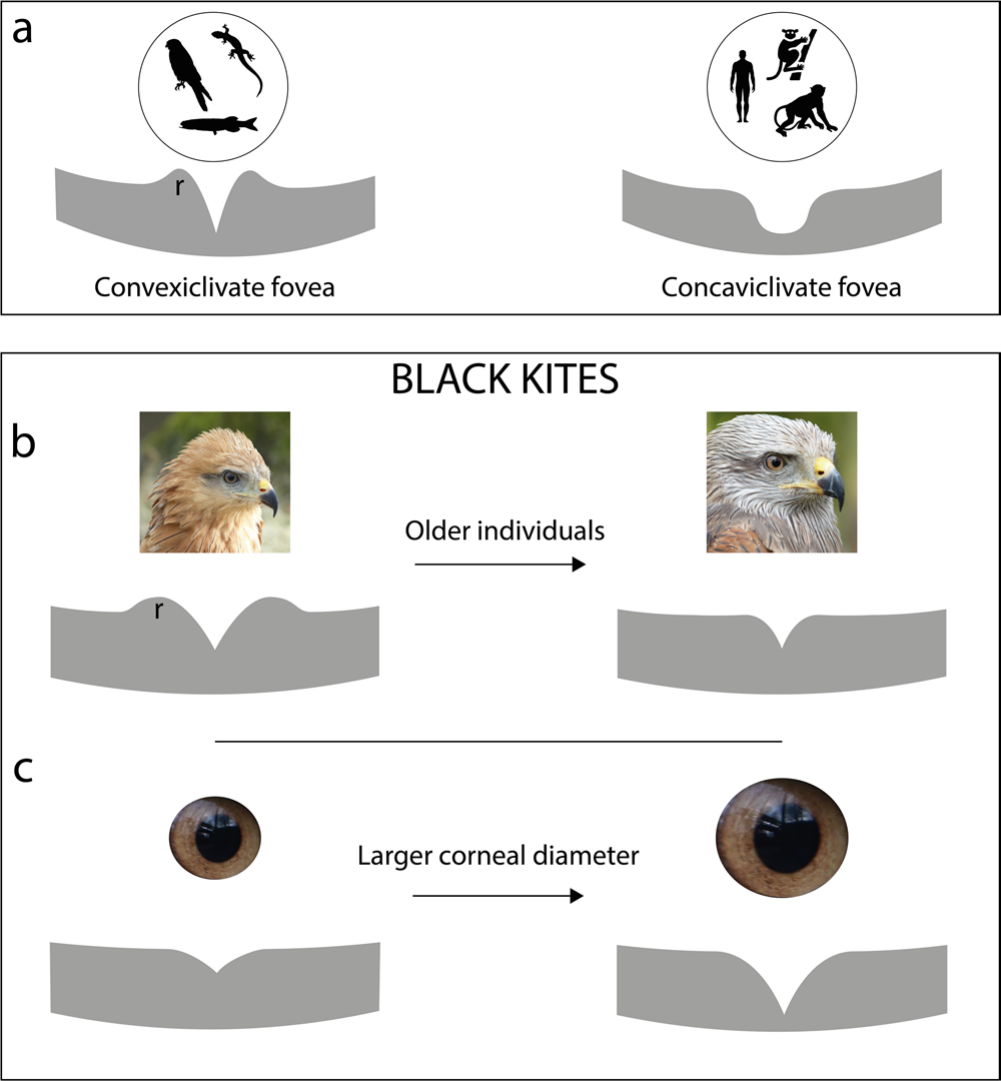
Schematic representation of the fovea(e). (**a**) Representation of the two foveae “types” as described by Walls (1942) with the convexiclivate fovea found in some birds, lizards or fish and the concaviclivate fovea found in some primates. (**b**) Black kite foveal shape depends on age. Younger birds had a deeper and wider fovea with more pronounced foveal rim (r). (**c**) Black kite foveal shape depends on corneal diameter. Birds with larger corneal diameter had a deeper and wider fovea. Because the depth increases more than the width, birds with larger corneal diameter had also steeper foveal slopes.

If “a bird is a wing guided by an eye” as Rochon-Duvigneaud^1^ wrote, it is clear that vision is an important sensory modality for birds, especially for those living in open environments where vision is not obstructed. Birds use their visual sense for many behaviours such as foraging, moving and mating^2^. In particular, birds of prey from the orders Accipitriformes and Falconiformes, hereafter called raptors, are believed to depend heavily on vision. Indeed, some raptors have the most acute vision found to date in the animal kingdom (see Mitkus, *et al*.^3^ for review). This acute vision is a result of having both relatively larger eyes than other birds^4,5^ and a well-developed fovea (some raptors have two foveae) with high densities of cone photoreceptors and ganglion cells^6–8^. A fovea is an invagination in the inner part of the retina that is specific to some primates, lizards, fish and many birds^9^. The central fovea of many birds, including raptors, along with some lizards and fishes, is deep and has been described as “convexiclivate”, as opposed to the shallower “concaviclivate” fovea found in primates (Fig. 1a)^9^.

The presence and physical structure of a fovea are thought to be linked to specific behaviours and foraging tactics. For example, in lizards, it has been shown that species with deeper fovea hunt for smaller prey^10^. In raptors, it has been shown that the majority of predatory species such as hawks and eagles have a second fovea placed temporally which is thought to be used for prey fixation at the moment of capture, while raptors that rely more heavily on scavenging, such as vultures and condors, lack this second fovea and instead possess an area of high photoreceptor and ganglion cells density called an area temporalis^11–13^. However, in general it is still unclear whether foveae with different shapes have functional differences. Indeed, the specific function of the fovea remains unresolved despite considerable attention since the 1940s^14–16^.

Some authors have suggested that the fovea may have an optical function. Walls^16^ and Locket^17^ suggested that the convex shape and steep slopes of the convexiclivate fovea combined with the difference between the refractive indices of the vitreous and retina may magnify the image. Snyder and Miller^18^, based on the examination of the central fovea from a hawk, suggested that strong magnification is only due to the concave bottom of the foveal pit, which could explain the high visual acuity of raptors compared to humans, despite the human eye being of equal size to that found in many raptors^7,8,19^. More recently, studies on foveal hypoplasia in humans showed that individuals either completely lacking a fovea, or with a less pronounced fovea, have poorer visual acuity^20,21^. However, it is unclear whether it is the structure of the fovea alone or associated retinal changes that reduce the visual acuity.

In contrast, others have stated that the physical structure of the fovea does not play a role for visual acuity. Pumphrey^22^, for example, considered that the magnification of the convexiclivate fovea should be uneven and distort the image of an object, except in the very centre of the fovea, thus facilitating visual fixation. A recent study found a slight magnification in the centre of the human concaviclivate fovea, but the effect appeared to be negligible^23^. It is desirable that such a study should be conducted on the convexiclivate fovea of birds. Theoretically, if the foveal shape had been selected for visual magnification, all “normal” individuals of a given species should have almost identical foveal shapes. However, in the “normal” population of humans, the foveal shape is highly variable^24^.

While studies on primate foveal shape have received growing interest in recent decades^15^, the function of the foveal shape of other animals remains poorly investigated. In raptors, the shape of the central fovea differs strongly among species^13,25^, but the reasons for this remain unclear. Moreover, Potier, *et al*.^13^ found that while foveal shape appeared to be consistent among individuals of many raptor species, this was not the case for one species in particular, the black kite *Milvus migrans*.

In this study, we build upon Potier *et al*.’s^13^ initial findings by identifying whether and how the foveal shape varied among a sample of more than 40 individual black kites of different age and gender, with different eye size and genetic proximity. One issue with many previous studies that have sought to describe foveal differences among individuals/species is that they have used parameters such as the depth, width (diameter) and/or gradients of the slopes^13,23,26^. Although useful measures, these parameters do not allow characterization of the overall shape of the fovea. Here, therefore, in addition to measuring the aforementioned parameters, we also used a geometric morphometric (GM) approach, *i.e*. a study of shape variation and its covariation with variables of interest that turns shapes into quantitative variables and then analyses variation between these variables^27–29^. Using this GM approach, it is possible to quantify and compare the overall foveal shape among individuals, independently of rotation, translation and rescaling^30^.

## Results

### Foveal size

The average corneal diameter of the black kites was 11.23 ± 0.04 mm (mean ± se; range: 10.31–12.32 mm) and did not differ between left and right eyes (Table 1). We found no differences in corneal diameter between males and females (Table 1), and no relationship between corneal diameter and the age of a bird (Table 1).

**Table 1:** Results from mixed models on classical measurements.

Measures	Effect	Estimate	Std.error	t	p
**Corneal diameter**					
	*Sex*	0.05	0.10	0.48	0.63
	*Age*	−5.69e-3	0.01	−0.37	0.71
	*Eye*	−0.06	0.05	−1.26	0.22
	Sex*Eye	−4.46e-3	0.10	−0.04	0.96
	Age*Eye	−0.02	0.02	−1.26	0.22
**Foveal depth**					
	*Corneal diameter*	22.62	9.40	2.37	**0.024**
	*Sex*	−6.84	6.31	−1.08	0.28
	*Age*	−2.34	1.02	−2.30	**0.026**
	*Eye*	5.40	5.86	0.92	0.36
	Corneal diameter*Eye	−13.66	18.71	−0.73	0.47
	Sex*Eye	−12.61	11.88	−1.06	0.30
	Age*Eye	0.03	1.96	0.01	0.99
**Foveal width**					
	*Corneal diameter*	45.56	13.84	3.29	**0.003**
	*Sex*	−11.83	15.79	−0.75	0.46
	*Age*	−19.63	2.51	−7.82	**<0.001**
	*Eye*	0.07	5.05	0.01	0.99
	Corneal diameter*Eye	10.15	16.75	0.61	0.55
	Sex*Eye	8.64	9.83	0.88	0.39
	Age*Eye	0.62	1.59	0.39	0.70
**Retinal thickness at the edge**					
	*Corneal diameter*	1.81	3.70	0.49	0.63
	*Sex*	0.03	3.71	8.55e-3	0.99
	*Age*	−0.57	0.57	−1.00	0.32
	*Eye*	0.99	1.32	0.75	0.46
	Corneal diameter*Eye	0.55	4.40	0.12	0.90
	Sex*Eye	4.35	2.56	1.70	0.10
	Age*Eye	−0.11	0.41	−0.26	0.80
**Retinal thickness at the rim**					
	*Corneal diameter*	0.97	5.42	0.18	0.86
	*Sex*	3.26	5.48	0.59	0.56
	*Age*	−2.20	0.87	−2.52	**0.015**
	*Eye*	1.08	1.88	0.57	0.57
	Corneal diameter*Eye	10.37	6.18	1.68	0.10
	Sex*Eye	1.91	3.54	0.54	0.59
	Age*Eye	0.86	0.56	1.52	0.14
**Retinal thickness at the pit**					
	*Corneal diameter*	−16.80	8.02	−2.09	**0.045**
	*Sex*	9.11	5.34	1.71	0.09
	*Age*	−0.07	0.85	−0.09	0.93
	*Eye*	−4.57	5.32	−0.86	0.40
	Corneal diameter*Eye	17.69	16.32	1.08	0.29
	Sex*Eye	16.00	10.67	1.50	0.14
	Age*Eye	0.23	1.72	0.14	0.89

**Figure 2:**
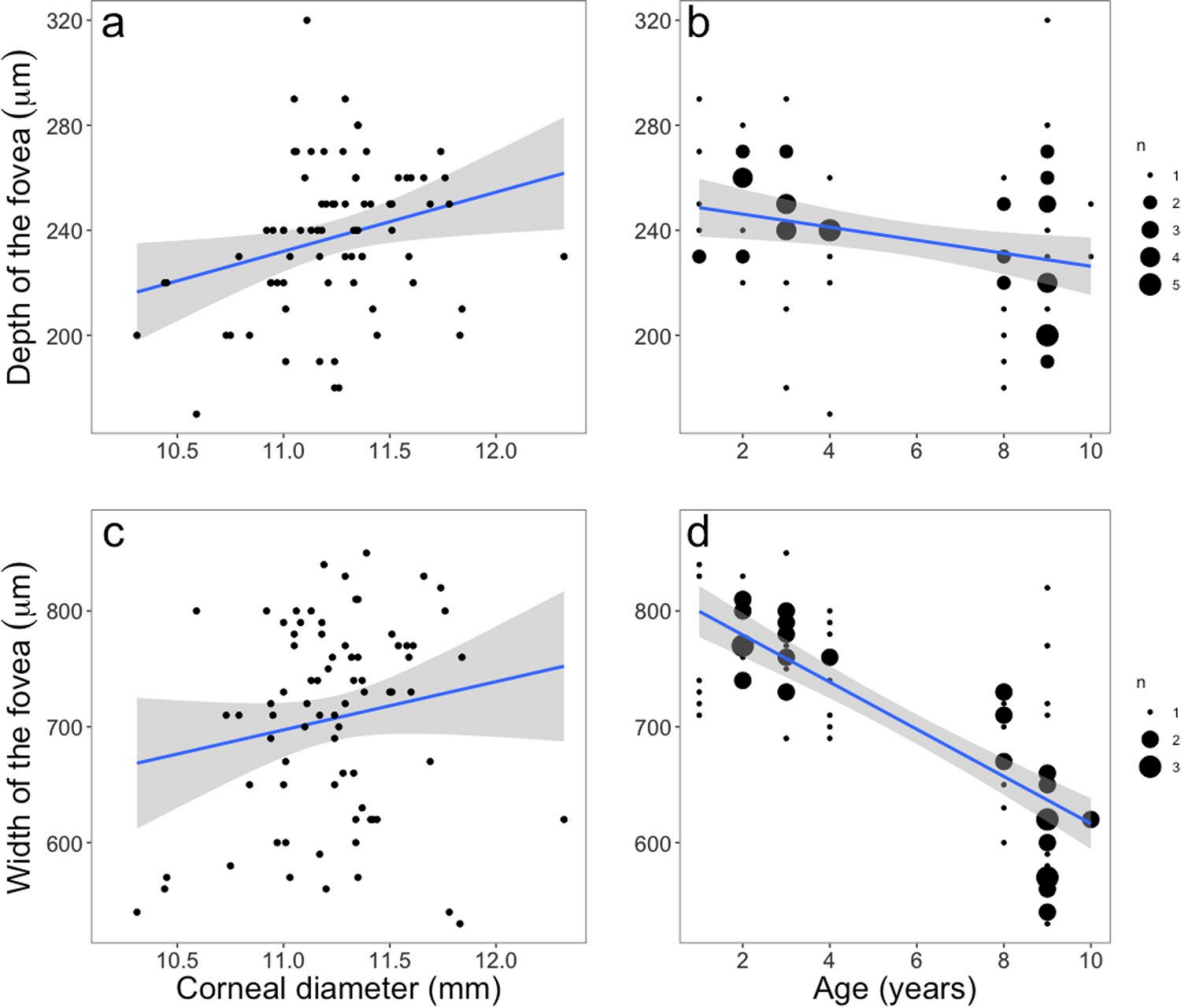
Relation between depth/width of the fovea and corneal diameter or age. Each dot represents a single eye, except for b and d where larger dots correspond to a higher number of eyes. Different size of dot represents the number of same value. Solid lines are mixed models predicted value and grey shades are SE (95%).

Birds with a larger corneal diameter had a deeper and wider fovea (Table 1, Fig. 2a,c), and younger individuals had a deeper and wider fovea than older birds (Table 1, Fig. 2b,d). This is true when we measure the full foveal depth including the foveal rim (all birds had a rim but of different thickness), which is the optically relevant measure. When foveal depth is calculated from the edge of the fovea, and not from the top of the rim, which may be more interesting from an anatomical point of view, the relationships between foveal depth and corneal diameter (Estimate = 18.81 ± 9.26, t = 2.03, p = 0.051) or foveal depth and age (Estimate = −0.42 ± 1.00, t = −0.42, p = 0.68) were not significant. Neither foveal depth nor width differed between sexes or between the left and right eyes (Table 1).

**Figure 3:**
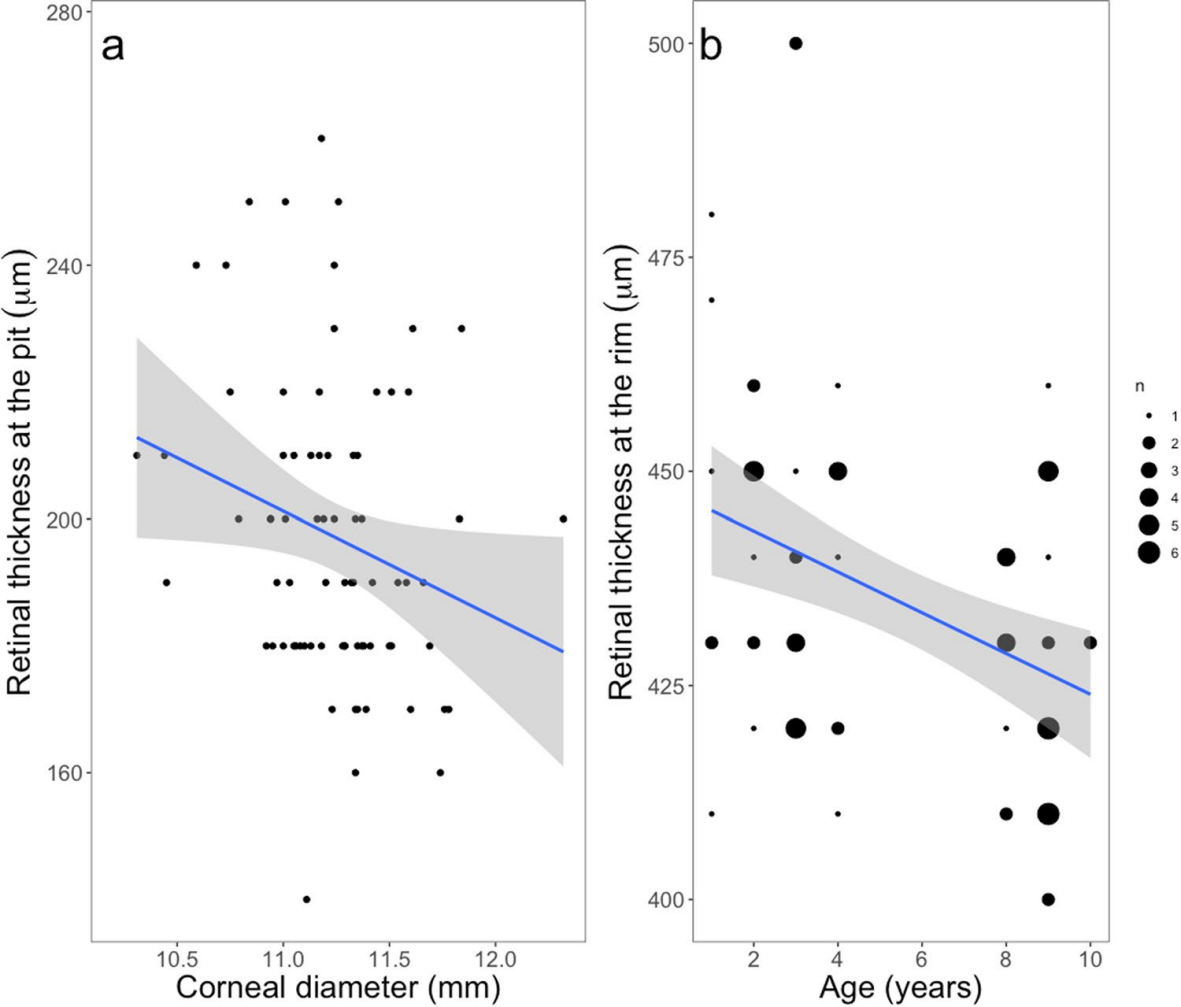
Relation between (**a**) retinal thickness at the pit and corneal diameter and (**b**) thickness at the foveal rim and age. Each dot represents a single eye, except for b where larger dots correspond to a higher number of eyes. Solid lines are mixed models predicted value and grey shades are SE (95%).

Retinal thickness at the foveal edge was not related to any variable of interest (Table 1). Independent of sex and corneal diameter, individuals had similar retinal thickness at the foveal rim, but younger individuals had a thicker retina at the foveal rim than older birds (Table 1, Fig. 3b, Fig. S1). Consequently, younger individuals had higher foveal rims than older birds (Estimate = −1.66 ± 0.64, t = −2.59, p = 0.013). The retinal thickness at the rim, as well as at the pit, did not differ between the left and right eyes of any individual. Retinal thickness at the pit also did not differ between individuals of different sex or age, but birds with larger corneal diameter had a thinner retina at the pit (Table 1, Fig. 3a).

No significant interactions between corneal diameter*eye, sex*eye, age*eye, were found for any measurements (Table 1).

### Geometric morphometric analyses of foveal shape

**Figure 4:**
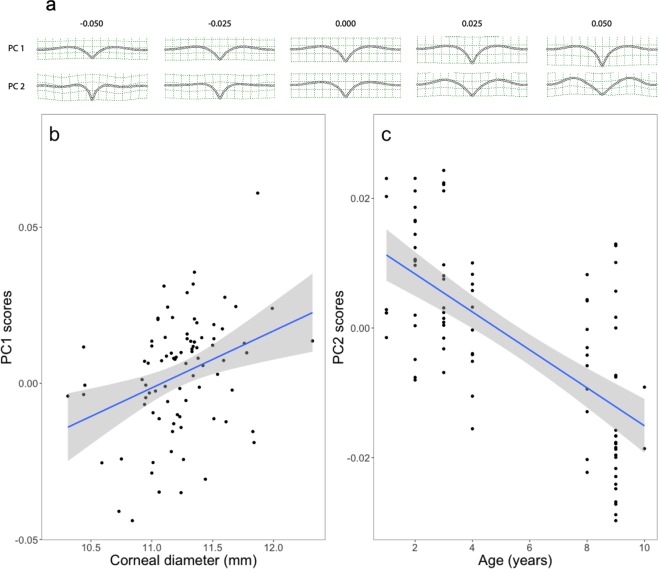
Results of Principal Component Analysis on shape data. (**a**) Aspects of the foveal shape captured by the first two components. (**b**) Relation between corneal diameter and PC1. (**c**) Relation between age and PC2.

The first two principal components (PCs) accounted for 76% of the total variance (48% for PC1 and 28% for PC2) and are discussed below. Positive PC1 scores represent steeper foveal slopes (elongation of the grid on the y-axis) while positive PC2 scores represent a wider fovea with more pronounced rim (Fig. 4a). Interactions between corneal diameter and eye (left or right) and between age and eye were not significant, for both PC scores (Table 2). While interaction between sex and eye for PC2 scores was not significant, we found a weak but significant interaction for PC1 scores (Table 2). However, after pairwise comparison, we only found a tendency that females had higher PC1 scores for the right eyes compared to their left eyes (t = −1.95, p = 0.059) or compared to the right eyes of males (t = 1.84, p = 0.074). All other comparisons were not significant (all p > 0.27).

**Table 2:** Results of mixed models from morphometric analyses.

Measures	Effect	Estimate	Std.error	t	p
**PC1 scores**					
	*Corneal diameter*	0.02	5.57e-3	3.27	**0.002**
	*Age*	−9.51e-4	5.93e-4	−1.60	0.12
	Corneal diameter*Eye	−0.02	0.01	−1.83	0.08
	Sex*Eye	−0.02	7.48e-3	−2.09	**0.044**
	Age*Eye	5.26e-5	1.18e-3	0.04	0.96
**PC2 scores**					
	*Corneal diameter*	3.59e-3	2.81e-3	1.28	0.21
	*Sex*	2.07e-3	3.13e-3	0.66	0.51
	*Age*	−2.88e-3	4.94e-4	−5.83	**<0.001**
	*Eye*	−1.53e-3	9.26e-4	−1.66	0.11
	Corneal diameter*Eye	3.89e-3	2.80e-3	1.39	0.17
	Sex*Eye	2.98e-3	1.79e-3	1.66	0.11
	Age*Eye	−5.41e-5	2.91e-5	−0.19	0.85

For PC1, we did not find any significant differences among individuals of different ages (Table 2). However, we found a positive relationship between corneal diameter and PC1 scores (Table 2, Fig. 4b). For PC2, we did not find any significant interaction (Table 2). We did not find any significant differences among individuals of different sex and corneal diameter, either (Table 2). However, we found a negative relation between PC2 scores and age (Table 2, Fig. 4c).

### Eye size, fovea, retina and genetic proximity

Genetic distance did not correlate with Euclidian distance in corneal diameter, foveal depth, foveal width, retinal thickness at the edge, rim and pit (Table 3). No significant relation between genetic proximity and PC1 or PC2 scores was found either (Table 3).

**Table 3:** Results of Mantel test to analyse relation between genetic proximity and Euclidian distance of all measurements.

Measures	Left eyes			Right eyes		
	rho	n	p	rho	n	p
Corneal diameter	0.01	21	0.42	−0.04	22	0.65
Foveal depth	0.11	17	0.14	−0.05	19	0.67
Foveal width	0.08	21	0.16	−0.06	22	0.78
Retinal thickness at the edge	0.02	21	0.38	−0.02	22	0.54
Retinal thickness at the rim	−0.05	21	0.71	−0.05	22	0.72
Retinal thickness at the pit	0.06	17	0.22	0.06	19	0.23
PC1 scores	−0.02	21	0.57	0.03	22	0.33
PC2 scores	0.01	21	0.41	0.06	22	0.21

## Discussion

The functional significance of foveal morphology remains unclear (see Bringmann^14^ and Bringmann *et al*.^15^ for reviews), and so exploring intraspecific variation in foveal shape within a population may help us to address this issue. Previous studies have estimated foveal shape using proxies such as depth and width, or estimation of the slopes of the foveal pit^13,23,26^. Here, we have used similar methods but also a powerful morphometric analysis, which allowed us to characterize any shape variation within our population. We propose that this approach is complementary to the analysis of depth and width. Indeed, while measurements of parameters such as foveal width and depth allowed us to estimate the exact size of the fovea, morphometrics add a new understanding of the shape variation.

In the black kites examined here, we found that birds with larger corneal diameter and thus, larger eyes, had a deeper and wider fovea when the rim was taken into account (Fig. 1c). Our morphometric analysis also revealed a stronger increase in depth compared to width (Figs. 1c; 4a,b) and, therefore, a steeper slope in individuals with larger eyes. A relationship between eye size and foveal depth in raptors has also been found at the inter-specific level^13^. If Walls’s^9^ magnification hypothesis is correct, individuals with larger eyes should have higher visual acuity not only because they have larger eyes, but also because of their steeper foveal slopes. Inter-individual variation in visual acuity has been found in another raptor species, the Harris’s hawk *Parabuteo unicinctus*^25^, but whether this is caused by a difference in foveal shape remains unknown.

A larger magnification effect of a fovea with steeper slopes may in part explain why visual acuity does not increase linearly with eye size^31^. If individuals with larger eyes have a fovea with steeper slopes, as is the case in black kites, and if the fovea magnifies the image, the spatial resolution should increase more strongly with eye size than expected by a linear regression. The magnification hypothesis is also supported by the fact that lizards hunting for larger prey have a shallower fovea than those hunting for small prey, *i.e*. at a same distance, they do not need as high a magnification as those hunting for small prey items^10^. However, such a magnification effect has still to be proved^22,23^. As mentioned previously, Frey *et al*.^23^ found a slight magnification in the centre of the human concaviclivate fovea, but the effect appeared to be negligible (although it would be interesting to perform a similar study on an avian convexiclivate fovea). Moreover, in a recent review, Bringmann^14^ suggested that there is no relationship between foveal depth and visual acuity in birds, and Reymond^7^ and Reymond^8^ found that behavioural visual acuity matched spatial resolution estimated from focal length and photoreceptor density in two raptor species, suggesting no magnification effect of the fovea.

It has also been suggested that variation in foveal shape could improve image fixation and sensitivity for movements, with better image fixation and sensitivity for movements in deeper fovea with steeper slopes^22^. According to this hypothesis, as individuals with larger eyes have a fovea with steeper slopes, they should have higher success in hunting fast-moving prey. However, there is no clear evidence supporting this hypothesis. For example, one could assume that predatory raptors need better fixation and movement detection abilities than scavengers, and therefore a deeper central fovea, yet Potier *et al*.^13^ found no such difference between accipitriform scavengers and predators. In contrast, however, the fact that a temporal fovea is only found in predatory species^11,13^ supports the suggestion that image fixation and sensitivity to movements may indeed be a function of the fovea. A behavioural experiment comparing the prey capture and/or prey detection abilities among individuals with different foveal shapes may be essential to test the hypothesis that a deeper fovea with steeper slopes aids prey fixation.

While age was not related to steeper foveal slopes in our black kites, younger individuals had a bigger fovea with a more pronounced rim than older birds (Fig. 1b). The development of the fovea has been studied in primates (see Bringmann, *et al*.^15^ for a review), but most studies have only explored variation in foveal morphology at an early stage of life. In the rhesus monkey *Macaca mulatta* for example, foveal depth decreased until an age of 150 days to reach 150.4 µm depth on average^32^. In a long-term study on the development of the human fovea (from 22nd week of gestation to a 72-year-old), Yuodelis and Hendrickson^33^ showed that the width of the rod-free zone decreased significantly with age, while cone density increased in the foveola (the central part of the fovea), indicating centripetal movement of photoreceptors in the macula.

In our study, the most important change, which correlated with the age of the birds, was the reduction of rim size at the foveal edge. This rim is formed by inner retinal cells that are centrifugally displaced from the foveal centre^15^. Therefore, if younger individuals have a more pronounced rim, this is likely caused by a higher number of ganglion cells and inner nuclear layer cells that are displaced centrifugally from the centre of the fovea. In pigeons, a loss of 33% of photoreceptors and 23% of ganglion cells in the area dorsalis has been observed between the age of 2 and 17 years^34^. If such loss occurs equally across the whole retina then it is possible that young black kites also have more photoreceptors and ganglion cells than adult birds, leading to the more pronounced rim at the edge of the fovea. This could also mean that younger individuals of black kites have a higher visual acuity than adults, as is the case in pigeons^34^ independent of sex.

Marked sex differences in visual system morphology and visual perception have been reported in a number of species. For instance, sexual dimorphism in eye size has been reported in fishes^35^ and reptiles^36,37^, while sex differences in visual perception have been found in humans^38^. In sagebrush lizards *Sceloporus graciosus* (Family: Phrynosomatidae), females are faster than males at visually detecting motion, which may be adaptive because it allows for better detection of complex courtship signals of males^39^. Phrynosomatid lizards possess a fovea^40^ and so following Pumphrey’s^22^ hypothesis that a fovea with steeper slopes allows for better motion detection, one could expect differences in foveal shape between males and females. There is currently limited information on the potential for sexual differences in vision that may also occur in birds. For instance, in the retina of European starlings *Sturnus vulgaris*, no difference in the number and types of cones have been found between sexes^41,42^. Furthermore, male and female brown-headed cowbirds *Molothrus ater* have similar eye size^43^. In our study, we did not find any difference in foveal shape between male and female black kites. Contrary to most raptors, in which females are larger than males, this is not obvious in black kites^44^. While in other species males and females select different prey item based on their size difference^45^, there is no evidence of such differences in black kites. Therefore, based on foraging, black kites of both sexes likely have similar visual demands.

We did not find any significant difference in foveal shape between the left and right eyes. Many birds exhibit a high degree of behavioural lateralisation, using a preferred eye for a variety of behaviours, such as viewing novel or familiar stimuli and food items, and for predator surveillance^42,46–48^. Moreover, in some species, significant differences in retinal organisation between the left and right eyes have been reported. For instance, the number of cones and/or ganglion cells differs between the left and right eye in European starlings^42^ and in cockatoos^46^. In falcons that use their fovea to fixate and pursue prey, no preference for the left or right eye has been found^49^. However, behavioural lateralization remains to be explored in black kites, which are preferentially scavengers and belong to a separate phylogenetic group (Accipitriformes) compared to falcons, which are falconiforms^50^.

Phylogenetic relatedness has been suggested to be an important factor in determining both the presence or absence of a fovea, as well as its shape^40^. Most bird species with a fovea have the “convexiclivate” type^9^, but whether the shape of this fovea is a heritable trait within a species remained unknown. In humans, heritability has been shown in some complex visual traits, such as refractive errors^51,52^. In birds, only little evidence for heritable visual traits has been found. For instance, axial length, vitreous chamber depth, corneal curvature and eye weight are highly heritable in chickens^53^. In our study, we found no relationship between corneal diameter or foveal shape and genetic proximity. All birds used in this study were captive birds belonging to the same park and raised under similar conditions, thus environmental conditions cannot be the reason for differences. Using the same population of black kites, we previously showed a significant and positive correlation between genetic proximity and chemical profiles, with more closely-related individuals having more similar preen oil chemical substances, suggesting a strong genetic determinism in this sensory trait in this species^54^. Further studies are needed to understand the link between genetic relatedness and other properties of the visual system.

## Conclusion

Most studies on the fovea of birds (and indeed other animals) have used very few individuals in their analyses (see Bringmann^14^ for a review). Our study revealed that foveal shape can be highly variable among individuals of the same species. Furthermore, this work provides evidence that corneal diameter and age are two important factors that should be taken into account in further analyses of avian foveae. For example, because eye size is positively correlated to prey capture technique in birds, with species hunting for actively moving prey having larger eyes^55^ and foveal depth is negatively correlated to prey size, at least in lizards^10^, black kites with large eyes and steeper-sloped foveae may have different foraging strategies than those with smaller eyes. We also found that younger individuals had a different foveal shape than older birds, with a larger rim suggesting that more ganglion and inner nuclear cells were displaced compared to older birds. Therefore, younger individuals could have more cones in their foveae than adults, and thus potentially a higher spatial resolution^56^ (but see Coimbra *et al*.^57^). If this was the case, younger individuals should have better visual abilities in relation to prey detection, which may compensate in part for their slow acquisition of hunting skills^58^. The black kite is an opportunistic raptor with a diversified diet^59^, and it would be very interesting to explore whether diet and foraging strategies differ among individuals with different age and eye size, and in relation to differences in their visual systems.

## Material and Methods

### Animals

The subjects were 47 black kites (24 females and 23 males) aged from 1 to 10 years that belong to the French falconry park “Le Grand Parc du Puy du Fou”. The birds are kept in good health and fly regularly. They were measured close to their holding aviaries and returned to them immediately after data collection.

### Corneal diameter

**Figure 5:**
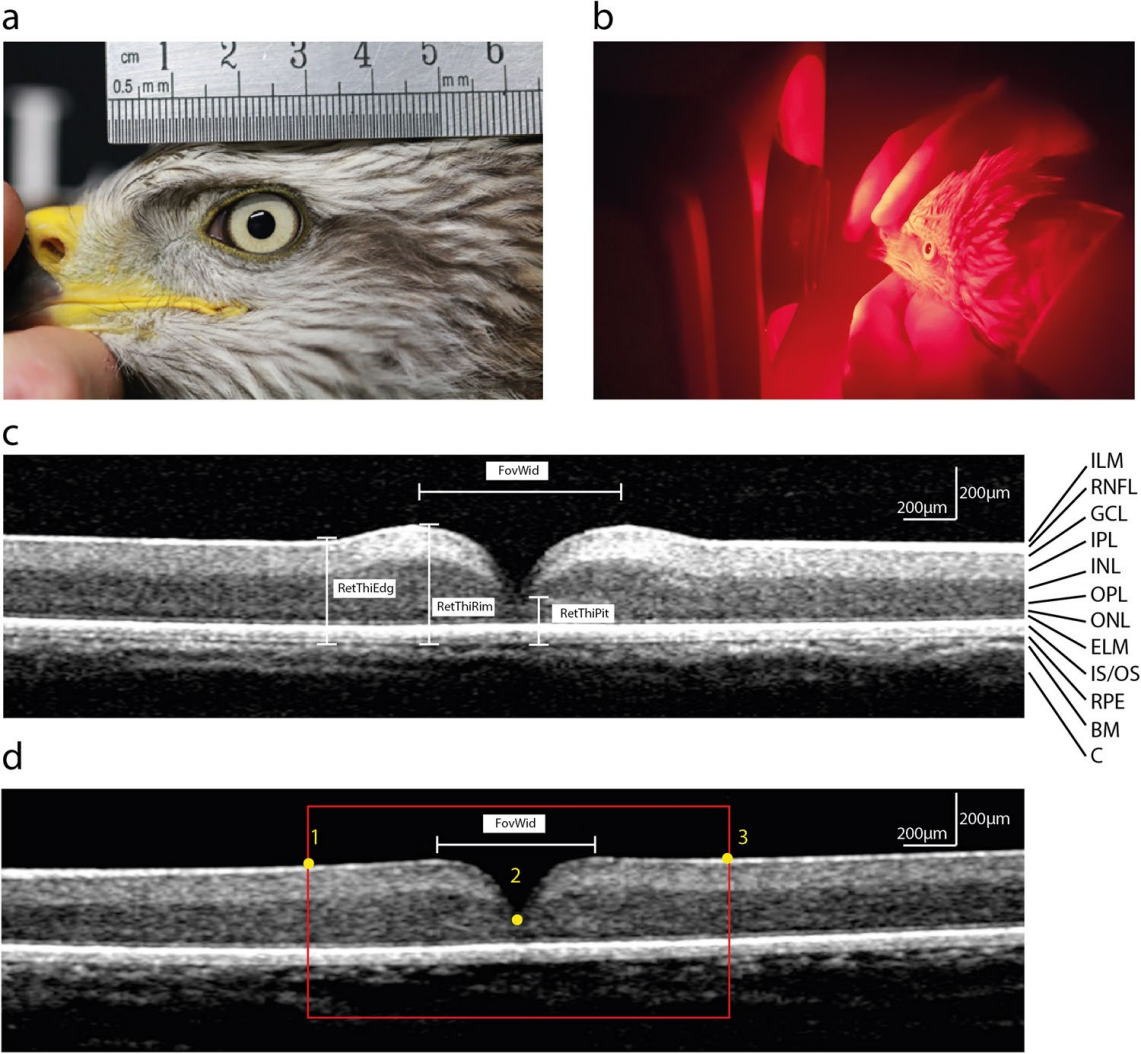
Experimental analysis of fovea. (**a**) Method used to measure the corneal diameter. (**b**) A black kite handled in the hand of the experimenter (S.P.) for SD-OCT measurements.(**c**,**d**) SD-OCT images (B-scans) of the central fovea of two black kites of different age (c: 2 years old; d: 7 years old) showing the foveal parameters used in classical analysis: retinal thickness at foveal edge (RetThiEdg), foveal rim (RetThiRim) and foveal pit (RetThiPit) as well as foveal width (FovWid). The red square represents the analysed zone for the geometric morphometric analysis (1600 µm in width). Homologous points (see materials and methods section) are represented in yellow in their specific order. Retinal layers: ILM, inner limiting membrane; RNFL, retinal nerve fiber layer; GCL, ganglion cell layer; IPL, inner plexiform layer; INL, inner nuclear layer; OPL, outer plexiform layer; ONL, outer nuclear layer; ELM, external limiting membrane; IS/OS, photoreceptor inner/outer segments; RPE, retinal pigment epithelium; BM, Bruch’s membrane; C, choroid.

We used corneal diameter as a proxy for eye size (Potier *et al*.^13^ and see below), measured using close-up photography. While a falconer handled the bird, a ruler was placed above the eye at the same distance to the camera (Fig. 5), and a close-up picture was taken with a camera (Canon D70, 55 mm Canon EF lens) at a 90° angle to the plane of the pupil. Using ImageJ (Rasband, 1997–2012), transversal corneal diameters were measured as the distance between the corneoscleral junctions on the nasal and temporal sides of the pupil. Both eyes were examined in all birds. The precision of this method has been tested before^13^. However, we removed the pictures of three eyes from the analysis because photographs were not taken at a 90° angle.

### SD-OCT image acquisition

We acquired retinal images using Spectral Domain Optical Coherence Tomography (SD-OCT). This non-invasive imaging technique, based on low-coherence interferometry, provides high-resolution cross-sectional images of biological materials^60^. We used a Spectral OCT/Scanning Laser Ophthalmoscopy (SLO) (OPKO/OTI, Inc., Miami, FL, USA) with an inbuilt super luminescent diode with a peak wavelength of 830 nm as a light source and obtained cross-sectional retinal images (B-scans) using the Line Scan mode (512 A-scans/B-scan, 27,000 A-scans/s). Birds were awake and alert during the entire imaging process, which took less than 15 min per individual. The birds were held gently by a falconer (S.P.), at the suitable distance between the objective lens and the eye to obtain a focussed image of the retina (Fig. 5). During the imaging process, the SLO function of the Spectral OCT/SLO device provided an en face “live” image of the eye fundus, which allowed us to locate the fovea. Each cross-sectional B-scan was coupled with its en face SLO fundus image, thus providing the exact position of the B-scan in the retina. We selected the B-scan image that fell directly on the centre of the fovea to perform the analysis. We imaged both eyes in every individual bird. However, we excluded some images from our analysis due to low image quality. In total, images from 86 foveae from 47 individuals were analysed.

The Spectral OCT/SLO device and objective lens used in this study are designed and calibrated for investigation of human eyes. The axial lengths of adult humans and black kite eyes are similar (20–35 mm^61^ and 20 mm^62^, respectively) and refractive indices of human and avian retinae and ocular media are also similar^63^. Thus, as opposed to what would be needed for the investigation of much smaller or much larger eyes, or eyes with very different refractive properties, we did not adjust the hardware or software settings. Therefore, as in previous OCT studies on raptor eyes^13,60^, we did not recalculate the axial scale of the OCT B-scans. Moreover, as axial scaling in OCT images is independent of ocular magnification^64^, it was possible to directly measure the foveal depth and retinal thickness based on the axial scale included by software in each B-scan image (Fig. 5). However, as transverse measurements in OCT images depend on the anterior focal length (or posterior nodal distance - PND) of the eye^64^, and thus on the retinal magnification factor (RMF), we rescaled the images of each eye individually. To obtain the RMF, we calculated the PND of each black kite eye based on the corneal diameter: PND ratio of accipitriform eyes (0.78 ± 0.01 (mean ± se), 6 species, 1–3 eyes per species; MinM and AK unpublished data). Then, to obtain the transversal scale for each image, we multiplied the RMF by the scan angle of the Spectral OCT/SLO device (29.7 degrees). Finally, as the Spectral OCT/SLO has an axial resolution of <10 μm (resolution in the A-scan), we rounded each foveal depth and retinal thickness measurement to the nearest 10 μm.

### Foveal measurements

We first determined that all foveae had a rim (also called a shoulder; Fig. 1), according to Potier, *et al*.^13^. For thickness measurements we defined the edge of the fovea when retinal thickness reaches that of the adjacent retinal regions (Fig. 5).

We measured retinal thickness at the edge of the fovea (RetThiEdg), retinal thickness at the foveal rim (RetThiRim) and retinal thickness at the centre of the pit (RetThiPit) from the vitreal surface of the retina to the outer edge of the retinal pigment epithelium (Bruch’s membrane; Fig. 5). Foveal depth (FovDep) was calculated relative to the retinal thickness at the foveal rim as FovDepRim = RetThiRim – RetThiPit, see Potier, *et al*.^13^. We also calculated foveal depth measurement relative to the retinal thickness at the edge of the fovea as FovDepEdg = RetThiEdg – RetThiPit. Rim thickness was calculated as RimThi = RetThiRim – RetThiEdg. Foveal width (FovWid) was calculated from the position where the slope of the pit ends, *i.e*. between the tops of the rim (Fig. 5c,d). Retinal layers were identified according to Ruggeri, *et al*.^60^.

### Geometric morphometric methods

Tpsdig software^65^ was used to set the coordinates of each landmark for each foveal image. First, we digitized three anatomical landmarks, one at the foveal pit and two located 800 µm on either side of the foveal pit, see Fig. 5c). Then, overall shape was quantified using 80 equidistant sliding semi-landmarks following the edge of the retina. TpsUtil software^66^ was used to create the sliding file and TpsRelw software^67^ was used to perform a General Procruste Analysis^30^ while allowing sliding semi-landmarks to slide minimizing the bending energy between the specimen and the mean shape^68^. All shapes were analyzed using a Principal Component Analysis (PCA).

### Genetic proximity

We used diversity at microsatellite loci as a proxy of genome-wide diversity for younger individuals (unfortunately, these data were not available for older birds, thus only 26 birds up to four years old were used for this part of study). Genetic analyses were conducted by the Labofarm laboratory (Loudéac, France). Individual genotyping data were taken from Potier, *et al*.^54^ based on 15 polymorphic microsatellite markers. For details on Genomic DNA extraction, amplification and determination of allele size, please see Potier, *et al*.^54^.

Genetic relatedness between individuals was estimated using the relatedness index ‘identity’ (*R*_ID_)^69^ calculated with the IDENTIX V1.1.5.0 software^70^. This index has been validated as a good estimator of the consanguinity of offspring in cases, in which alleles are likely to be identical by descent^70^.

### Statistics

All statistical analyses were performed with R 3.5.1 using *vegan*^71^, *ggplot2*^72^, *RVAideMemoire*^73^, *nlme*^74^, *gridextra*^75^, *emmeans*^76^ and *Matrix*^77^ packages.

To analyse differences in foveal shape in relation to eye size, gender, age and genetic proximity, we used two methods. First, using standard foveal measurements, we analysed the depth and the width of the fovea, as well as the retinal thickness at the edge, rim and pit of the fovea (see Potier *et al*.^13^ for such analyses). We used generalized linear mixed models (GLMMs) following Gaussian error distribution with foveal measurements (foveal depth, foveal width, retinal thickness at the edge, rim and pit) as dependent variables, corneal diameter, age, gender and eye (in interaction with all three other terms) as fixed effects, and individual as a random effect. Age was considered as a continuous variable, but similar results were found if considered as categorical variable with young individuals from 1–4 years and old individuals from 8–10 years (see Table S1 in Supplementary Materials).

In a second analysis, we used a morphometric approach to examine the overall shape of the fovea. Principle component (PC) scores from the PCA were utilized to visualize shape variations among individuals. We then performed GLMMs following Gaussian error distribution on PC scores using age, corneal diameter, gender and eye (in interaction with all three other terms) as a fixed effect and individual as a random effect. For all GLMMs, the best fixed effect structure was selected using AIC criterion following the method described by Zuur, *et al*.^78^. We inspected the residuals of each model to ensure that they fitted the assumption of GLMMs.

A Mantel test with Spearman rank correlation and 9999 permutations was used to test for an association between inter-individual Euclidian distance of corneal diameter, foveal depth, foveal width, retinal thickness at the edge, rim and pit and genetic proximity.

### Ethical approval

The experiments were carried out in accordance with European Parliament directive 2010/63/UE mentioning the non-application of an ethical committee when non-invasive experiments are conducted on captive animals following good veterinary practice. During the entire image process, birds were handled by a qualified falconer (S.P. who has a “Certificat de capacité” n° DDPP-18-283) and have been under strict veterinary control (P-F.I.). Birds were also followed by a vet (P-F.I.) during 48 h after the experiment. Furthermore, in agreement with French law, birds were handled by their usual trainer, under the permit of the Grand Parc du Puy du Fou (national certificate to maintain birds “Certificat de capacité” delivered to the director of the falconry, Jean-Louis Liegeois on April 7, 1994). The experiment has been reviewed and approved by the director of the falconry and the Grand Parc du Puy du Fou under the permit n°09-DRCTAJE/1-461.

## Supplementary information

Supplementry information.

Supplementry information 2.

## Data Availability

Data have been uploaded as Supplementary Material.
