# Supplementary material for: Inter-individual differences in foveal shape in a scavenging raptor, the black kite Milvus migrans
Source: Sci Rep. 2020 Apr 9;10:6133. doi: 10.1038/s41598-020-63039-y (PMC7145841; doi:10.1038/s41598-020-63039-y)
Supplement: Supplementary file 2 — Supplementry information 2. [file 41598_2020_63039_MOESM2_ESM.pdf]

Supplementary materials: Inter-individual differences in foveal shape in a scavenging raptor, the black kite *Milvus migrans*

Simon Potier[1*], Mindaugas Mitkus[1,2], Thomas J. Lisney[3], Pierre-François Isard[4], Thomas Dulaurent[4], Marielle Mentek[4], Raphaël Cornette[5], David Schikorski[6] and Almut Kelber[1]

1 Department of Biology, Lund University, Sölvegatan 35, Lund S-22362, Sweden

2 Institute of Biosciences, Life Sciences Center, Vilnius University, Lithuania

3 CEFE UMR 5175, CNRS - Université de Montpellier - Université Paul-Valéry Montpellier - EPHE, Montpellier, France

4 Unité d'Ophtalmologie, Centre Hospitalier Vétérinaire, Saint-Martin-Bellevue, France

5 Institut de Systématique, Evolution, Biodiversité (ISYEB) - Muséum National d'Histoire Naturelle, CNRS, Sorbonne Université, EPHE, Université des Antilles, 57 rue Cuvier, CP 50, 75005 Paris, France

6 Laboratoire Labofarm, Loudeac, France

[*]corresponding author: sim.potier@gmail.com

Table S1: Results from mixed models on classical measurements with age as a categorical variable (young birds: 1-4 years old; old birds: 8-10 years old).

Measures	Effect	Estimate	Std.error	t	p
Corneal diameter	Age	0.03	0.10	0.28	0.78
	Age*Eye	-0.03	0.11	-0.28	0.78
Foveal depth	Age	13.67	6.48	2.11	**0.041**
	Age*Eye	2.80	12.38	0.23	0.82
Foveal width	Age	123.77	16.05	7.71	**<0.001**
	Age*Eye	-3.59	9.95	-0.36	0.72
Retinal thickness at the edge	Age	3.77	3.63	1.04	0.30
	Age*Eye	1.02	2.59	0.39	0.70
Retinal thickness at the rim	Age	13.03	5.58	2.33	**0.024**
	Age*Eye	-5.07	3.56	-1.42	0.17
Retinal thickness at the pit	Age	1.23	5.39	0.23	0.82
	Age*Eye	-4.83	10.83	-0.44	0.66

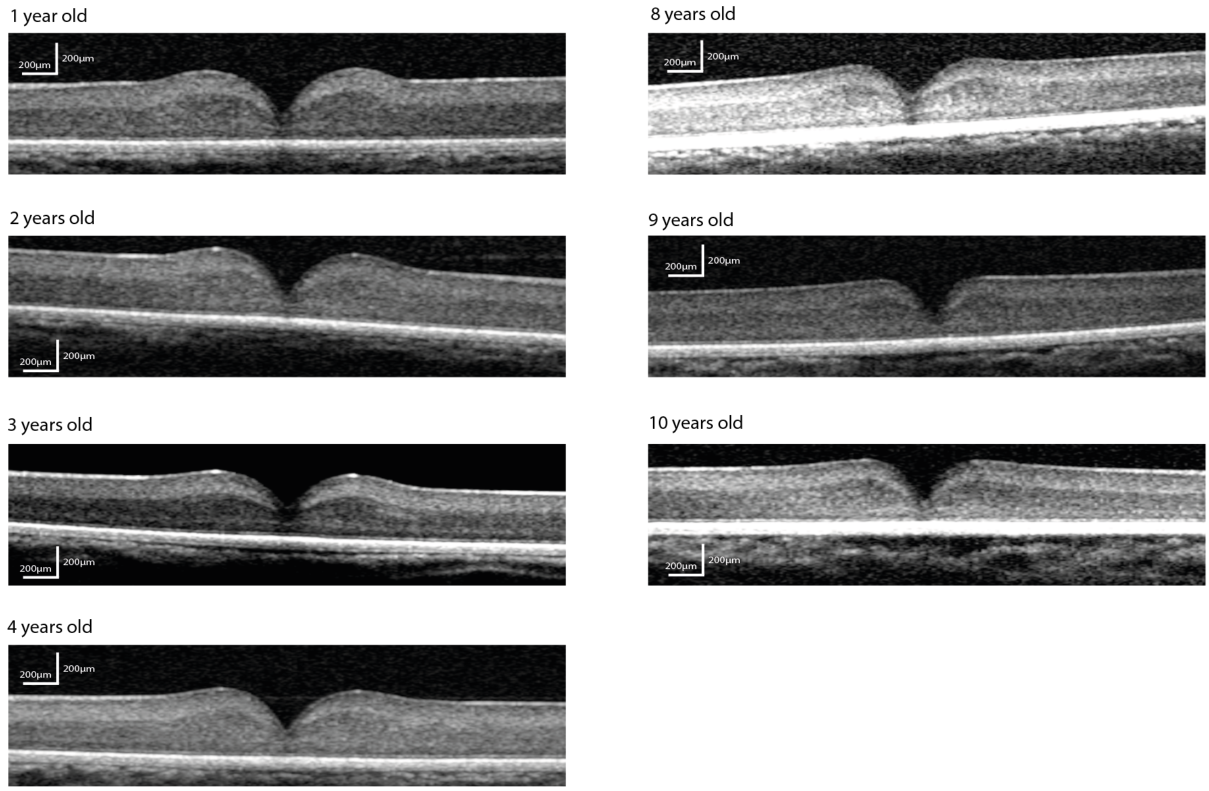

Figure S1: SD-OCT images (B-scans) of the central fovea of black kites of different ages.
